# Iatrogenic thoracic inlet narrowing during growing rod distraction causing critical tracheal stenosis: a case report and management implications

**DOI:** 10.1186/s12893-026-03881-5

**Published:** 2026-06-01

**Authors:** Shuqi Sun, Song Li, Zhen Liu, Benlong Shi, Yong Qiu, Zezhang Zhu, Saihu Mao

**Affiliations:** Division of Spine Surgery, Department of Orthopedic Surgery, Nanjing Drum Tower Hospital, The Affiliated Hospital of Nanjing University Medical School, Zhongshan Road 321, Nanjing, 210008 China

**Keywords:** Tracheal stenosis, Thoracic inlet, Thoracic lordosis, Congenital kyphoscoliosis

## Abstract

**Background:**

Respiratory complications in pediatric spinal deformities are most commonly attributed to thoracic insufficiency syndrome resulting from reduced thoracic volume and impaired chest wall compliance. Severe airway compromise caused by extrinsic tracheal compression is rare and remains under-recognized. Here, we report a rare case of extrinsic tracheal compression caused by progressive upper thoracic hyperlordosis after successive growing-rod interventions.

**Case presentation:**

A 6-year-old girl with progressive thoracolumbar kyphoscoliosis and compensatory upper thoracic lordosis (straight back) underwent halo-gravity traction and dual growing rod insertion surgery uneventfully, achieving considerable curve correction. In the subsequent first distraction surgery one year later, she developed airway spasm and hypoxemia during anesthetic intubation and encountered substantial difficulty in extubation postoperatively. Subsequent imaging and bronchoscopy showed severe tracheal narrowing at the thoracic inlet due to extrinsic compression. Initial attempts of rod adjustment provided little improvement. After thorough literature review and carefully weighing the pros and cons of various methods, a third posterior spinal procedure with multi-level posterior column osteotomies (PCOs) and kyphotic-contoured rods successfully restored upper thoracic kyphosis, widening the thoracic inlet and trachea. She was successfully extubated on postoperative day two and remained respiratory asymptomatic with stable deformity correction at 1-year follow-up.

**Conclusions:**

This case highlights that the accompanying upper thoracic hyperlordosis (straight back) in pediatric spinal deformity may cause thoracic inlet narrowing and occult asymptomatic airway compression which may become critically aggravated after distraction-based growing rod surgery. Posterior restoration of upper thoracic kyphosis may be an effective and less invasive strategy for relieving tracheal compression in selected cases.

## Background

Studies on respiratory disorders associated with pediatric spinal deformities have mostly focused on restrictive or obstructive ventilatory impairments resulting from a reduced, asymmetrical, and distorted thoracic cavity with decreased chest wall compliance, and the most severe manifestation is known as thoracic insufficiency syndrome [1–4]. The literature remains limited concerning respiratory dysfunction in scoliosis/kyphoscoliosis surgery caused by severe airway stenosis [5, 6]. Occult narrowed sternospinal channel caused by straight back syndrome was reported to be a significant cause for unanticipated severe airway events during anesthesia and surgery [5, 7–9]. If such upper thoracic hyperlordosis is associated with clinically silent tracheal narrowing, the airway hazards tend to be easily overlooked in clinical practice and may result in catastrophic consequences. Despite being surgically correctable, the salvage treatment strategies are usually complex, individualized and technically demanding [10]. Here we report a pediatric patient who encountered unanticipated severe tracheal stenosis caused by progressive thoracic inlet narrowing associated with increasing upper thoracic lordosis after growing-rod insertion and subsequent distraction procedures for correction of spinal deformity. The treatment process was quite tortuous and complicated, and the lessons learned suggested that airway assessment was essential for patients with upper thoracic hyperlordosis during spinal deformity surgery.

### Case presentation

**Fig. 1:**
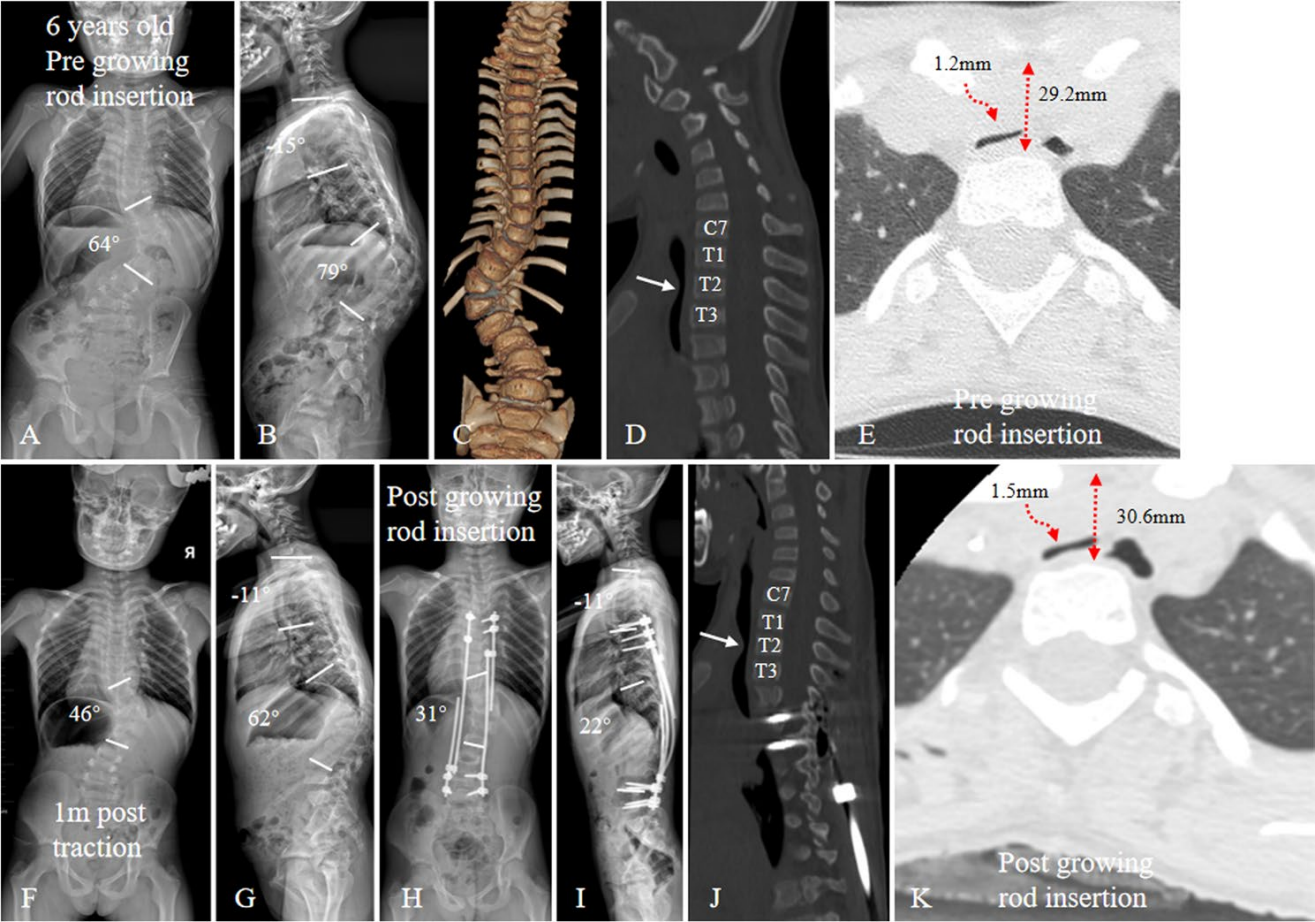
(**A-B**) Anteroposterior and lateral X-ray films showed severe thoracolumbar kyphoscoliosis along with compensatory thoracic lordosis. The C7-T5 angle on the lateral radiograph was − 15°. (**C**) Preoperative CT reconstruction showed significant wedging vertebrae at the apical area. (**D-E**) Preoperative CT scan revealed a narrowed thoracic inlet (AP diameter: 29.2 mm) with moderate tracheal stenosis (AP diameter: 1.2 mm) (T2 level). (**F-G**) After 1 month of halo-gravity traction, the scoliosis and kyphosis improved to 46° and 62°, respectively, and the C7-T5 angle improved slightly to -11°. (H-K) After growing rod insertion, the deformities were significantly corrected, while the C7-T5 angle remained − 11°. The anteroposterior diameters of both the thoracic inlet and the previously compressed airway increased slightly postoperatively (30.6 mm and 1.5 mm, respectively)

A 6-year-old girl was admitted for corrective surgery due to progressive back protrusion over a 3-year period. Radiographic examination revealed thoracolumbar kyphoscoliosis associated with compensatory upper thoracic hyperlordosis (straight back, C7-T5 sagittal angle: -15°) (Fig. 1A-B). Significant vertebral wedging of T12-L1 and vertebral dysplasia on the left side was observed on 3D computed tomography (CT). The whole spine MRI scans showed no spinal cord malformation. The deformity was considered congenital in origin.

A summary of the whole treatment procedures is presented in Table 1. Initially, the child was treated with halo-gravity traction for one month before growing rod insertion surgery. After traction, the thoracolumbar curve and kyphosis improved from 64° and 79° to 46° and 62°, respectively. The C7-T5 angle improved slightly to -11° after traction (Fig. 1G). A traditional dual growing rod construct was then inserted uneventfully, with the proximal and distal anchors placed at T5-T7 and L4-L5, respectively. Postoperatively, the scoliosis and kyphosis were corrected to 31° and 22°, respectively.

**Table 1:** Chronological summary of the clinical course, interventions, and airway-related outcomes

Time point	Clinical status / finding	Intervention	Outcome
Initial presentation	Progressive thoracolumbar kyphoscoliosis with compensatory thoracic lordosis; no dyspnea or other obvious respiratory symptoms	Halo-gravity traction for 1 month	Considerable correction of scoliosis and kyphosis
After traction	Persistent deformity requiring growth-friendly treatment	Initial dual growing-rod insertion	Deformity improved; no perioperative airway event
1 year later	Curve progression; severe mixed ventilatory dysfunction on pulmonary function testing	First planned growing-rod distraction	Airway spasm, wheezing, and hypoxemia after intubation; multiple attempts at extubation failed
Post-distraction airway evaluation	Bronchoscopy and CT showed severe tracheal narrowing at the thoracic inlet; retrospective review showed that moderate narrowing had already been present preoperatively	Bronchoscopy, CT, and retrospective imaging review	Extrinsic compressive tracheal stenosis recognized
First revision attempt	Persistent airway compromise	Rod release and proximal in situ bending	Minimal airway improvement; extubation still failed
Second revision (definitive)	Critical airway stenosis requiring urgent relief	Multilevel PCOs (C7-T5) and kyphotic-contoured rods	Restoration of upper thoracic kyphosis with marked widening of the thoracic inlet and trachea; successful extubation
1-year follow-up	No recurrent dyspnea	Clinical and radiographic follow-up	Stable deformity correction and maintained airway patency

**Fig. 2:**
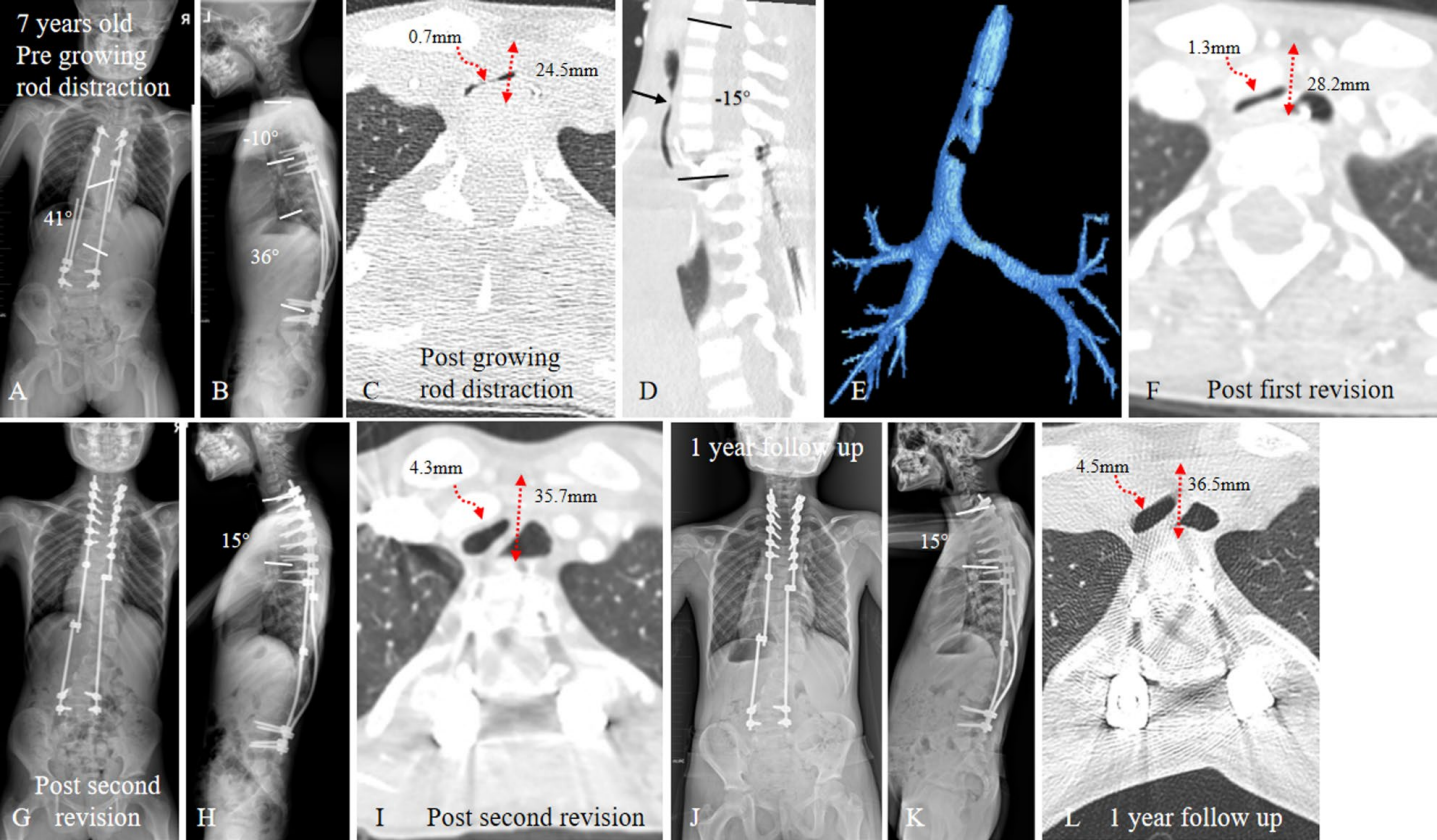
(**A-B**) The coronal balance slightly inclined to the convexity before the first growing rod distraction surgery, and the C7-T5 angle was − 10°. (**C-E**) After growing rod distraction, marked tracheal collapse (AP diameter: 0.7 mm) occurred with significant narrowing of thoracic inlet (24.5 mm), and the C7-T5 angle worsened to -15°. (**F**) After the first revision surgery, tracheal stenosis (AP diameter: 1.3 mm) improved mildly with a mildly enlarged thoracic inlet (28.2 mm). (**G-I**) After the second revision surgery, the AP diameters of trachea (4.3 mm) and thoracic inlet (35.7 mm) were markedly improved. The C7-T5 angle changed to 15°, indicating restoration of upper thoracic kyphosis. (**J-L**) At the 1-year follow-up, the tracheal patency was well maintained with good corrections of the deformities, and the C7-T5 angle remained 15°

One year later, the patient was hospitalized again for the first growing-rod distraction procedure. Radiographs demonstrated moderately progressed scoliosis (41°) and kyphosis (36°). Before distraction, the C7-T5 angle was − 10° (Fig. 2B). Preoperative pulmonary function testing demonstrated severe ventilatory dysfunction with predominant restriction. The forced vital capacity (FVC), forced expiratory volume in 1 s (FEV1) and FEV1/FVC were 47.3%, 48.3%, and 87.5% of predicted, respectively. After anesthesia induction and intubation, unexpected airway spasm, wheezing, and decreased blood oxygen saturation occurred. Emergency symptomatic treatment was applied to alleviate the wheezing and restore the oxygen saturation. The distraction surgery was quickly performed. After surgery, the first attempt to extubate failed. Although symptomatic treatment was continuously implemented, repeated attempts to extubate were still unsuccessful. Hospital consultation suggested a supplementary fiberoptic bronchoscopy examination. The results indicated that the tracheal lumen had narrowed to a slit approximately 5 cm distal to the tip of the endotracheal tube, corresponding to the T2 level. The chest CT scans and the airway reconstruction also demonstrated a significantly narrowed tracheal lumen at the thoracic inlet spinal regions (Fig. 2C-E). After distraction, the C7-T5 sagittal angle worsened to -15° (Fig. 2D). The site of obstruction corresponded to the thoracic inlet, where the trachea was confined between the anterior mediastinal structures and the anteriorly displaced upper thoracic spine. In this patient, the abnormal upper thoracic lordotic alignment reduced the AP dimension of the thoracic inlet and likely decreased the space between the trachea and the overlying innominate artery, resulting in extrinsic compression of the trachea. These findings prompted a careful retrospective review of the preoperative CT scans, which revealed moderate tracheal stenosis at the same level (Fig. 1D-E) before the initial growing-rod insertion, indicating that the distraction surgery aggravated a pre-existing airway abnormality rather than creating a completely new lesion. However, the clinical significance was overlooked at that time because the patient had no dyspnea or other respiratory symptoms in daily life, no airway-related event occurred during the initial surgery, and the surgeons lacked relevant experience. In addition, comparison of the preoperative and postoperative CT scans after the initial surgery showed no worsening of the narrowing (Fig. 1E, K), rendering the hidden airway risk inconspicuous.

Given that there had been no complaints of dyspnea before the distraction surgery and that anesthesia in the prone position carried current risks, we attempted to release the growing rod in the hope of restoring the preoperative status. In situ bending of proximal rods to move outward the upper instrumented vertebrae was also performed aiming to enlarge thoracic inlet indirectly. Intraoperative scanning with the O-arm navigation system was used to monitor airway diameter, revealing only minimal improvement. During incision closure, a critical episode of hypoxemia recurred, probably caused by thoracic inlet compression in the prone position. An emergency change of body position to the lateral decubitus position was performed to improve airway ventilation, restore oxygenation, and successfully complete the surgery. The postoperative attempt to extubate failed again due to the presence of the three depressions sign and hypercarbia, and the patient had to be reintubated. Contrast-enhanced CT was suggested, which revealed that the trachea was still flattened to a slit in a remarkably constricted space between the innominate artery and the anteriorly displaced vertebral column.

A second multidisciplinary consultation was arranged since an operation for relief was urgently warranted. The following treatment options were proposed: endoscopic tracheal stent implantation, anterior mediastinal tracheostomy or thoracotomy for airway decompression. After careful consideration of the risks and benefits, informed by the literature review, the final surgical strategy was to restore upper thoracic kyphosis through posterior spinal instrumentation in order to enlarge the thoracic inlet directly. For anesthesia preparation, a thinner and longer endotracheal tube was used under flexible bronchoscopic guidance to navigate the narrowed airway and permit prolonged prone-position surgery. A third posterior spinal operation was then performed, involving multi-level PCOs from C7 to T5 within the segments of thoracic lordosis. Kyphotic-contoured rods were inserted to elevate the screws and restore upper thoracic kyphosis, with the C7-T5 angle increasing to 15° after the second revision surgery (Fig. 2H). Intraoperatively, anatomic correction was immediately demonstrated on O-arm scans, showing a significant widening of the patient’s tracheal diameter and allowing the surgeons to confidently conclude the surgery. Successful extubation was achieved on the second day with full resolution of obstructive symptoms. At the 1-year follow-up, no recurrence of dyspnea was observed.

## Discussion and conclusions

Upper thoracic hyperlordosis and secondary thoracic inlet narrowing with tracheal stenosis have been reported in multiple conditions including connective tissue diseases, straight back syndrome, Pompe disease and cerebral palsy with opisthotonus [7, 9, 11, 12]. Interestingly, a considerable portion of critical airway events in the literature were caused by iatrogenic tracheal compression or stretching secondary to direct vertical compression of the chest in the prone surgical body position or to geometrical spinal shape changes after scoliosis correction surgery [8, 13]. Gradual aggravation of respiratory distress, however, was more frequently reported in those with motor and intellectual disabilities [7, 14]. Collectively, these reports suggest that airway compromise related to upper thoracic deformity may remain clinically silent before surgery but become critical during or after deformity correction. Chronic mechanical compression may also be associated with secondary tracheomalacia or, in rare cases, trachea-innominate artery fistula, especially in patients with connective tissue defects and weak tracheal tissue [15]. In the present case, although tracheomalacia was considered in the differential diagnosis, it appeared less likely because the airway demonstrated spontaneous and marked expansion after improvement of the thoracic inlet and upper mediastinal space.

In a few reported cases, airway obstruction was alleviated following respiratory support [8, 16]. In cases refractory to respiratory support, urgent surgical intervention was required. Several distinct surgical techniques, though limited in number, have been devised for the treatment of this rare condition. Although these techniques have achieved successful outcomes, the underlying mechanisms and the associated surgical trauma vary considerably. Intraluminal tracheal stenting has been favored to achieve palliation of the compression with minimal trauma. However, this technique was associated with mixed success, and concerns regarding the adverse effects in the long-term follow up including stent migration, infection, intraluminal formation of obstructive stent granuloma or erosion of the tracheal wall with fistula formation and fatal bleeding can’t be ignored [17–20]. Recently, downsizing biodegradable Ella stents rather than metallic or silicone stents have been applied for treating tracheobronchomalacia, which hold out the promise of reducing the complication rates [21]. Alternatively, temporary use of retrievable self-expanding metal stents was proposed by Narin to serve as a bridge to secure implementation of corrective surgery for spinal deformity, so as to mechanically reconstruct the superior mediastinum for restoring the airway patency permanently [6].

Regarding the anterior approach to create adequate substernal space for mechanical decompression, several tailored corrective techniques have been proposed involving sternoplasty, sternal division, partial claviculectomy, correction of severe pectus excavatum, transection or reimplantation of the innominate artery, anterior vertebral osteotomies and anterior mediastinal tracheostomy [10, 14, 22–25]. Despite their complexity and diversity, the underlying mechanisms involved resection of the anterior bony structures of the thoracic inlet; repositioning and reconstruction of the central vessels (transection, reimplantation, or sling); mirror transposition of the trachea combined with tracheostomy; and, finally, tracheopexy and tracheoplasty. Given that all of these approaches are invasive, technically challenging, and unfamiliar to spine surgeons who rarely encounter such unique anatomical conditions, less invasive and more readily implementable techniques are preferable whenever feasible. In particular, when the airway compromise is driven primarily by spinal alignment rather than intrinsic tracheal disease, correction of the spine itself may be a more logical solution.

From the perspective of spine surgeons, reversing the straightened or hyperlordotic spine to a kyphotic profile is a key step in recanalization of the trachea. This strategy achieves dual objectives while avoiding further surgical trauma to the anterior structures within the superior mediastinum. The retrodisplacement of the protruding vertebrae following sufficient reconstruction of upper thoracic kyphosis will create adequate mediastinal space and improve the airway patency spontaneously. In the literature, one patient with late-onset Pompe disease with undiagnosed critical tracheal stenosis [7] and two patients with cerebral palsy, cervicothoracic hyperlordosis and diagnosed tracheal stenosis [12] were weaned from respiratory support successfully after simple posterior spinal fusion. Given these precedents, the advantages of this strategy lie in that the related techniques involving PCO and vertebrae suspension technique using kyphotic-contoured rods were familiar to spine surgeons, making the management more accessible.

In conclusion, iatrogenic central airway obstruction in the setting of hyperlordosis-associated thoracic inlet narrowing may occur unexpectedly and requires prompt recognition and intervention. This rare case highlights the importance of careful preoperative respiratory and airway evaluation in spinal deformity patients with upper thoracic hyperlordosis, even when respiratory symptoms are absent or minimal. In addition, when growing-rod treatment is planned in pediatric patients, the proximal fixation anchors should preferably span across the hyperlordotic upper thoracic segments rather than terminate at or distally neighbouring the apex of upper thoracic hyperlordosis, based on the empirical lesson from this case. However, given the rarity of such cases, a quantitative comparative analysis to determine the relative contributions of spinal geometry, mediastinal confinement, and vascular factors to the airway narrowing was not feasible. All in all, based on empirical experience rather than evidence-based medicine, this rarely reported successfully rescued case indicated that extending posterior spinal instrumentation to the cervicothoracic junction and restoring a kyphotic upper thoracic profile provides an effective strategy to relieve tracheal compression without disturbing anterior mediastinal structures, thereby avoiding the risks of complex anterior remedial procedures. For extreme or refractory cases with persistent dyspnea, recurrent desaturation, or respiratory failure, additional anterior approaches can still be considered as a second-line option, but only after careful multidisciplinary evaluation.

## Data Availability

The datasets used during the current study are available from the corresponding author on reasonable request.

## Abbreviations

GR: Growing rod
PCO: Posterior column osteotomy
AP: Anteroposterior
CT: Computed tomography
